# Association Between Cardiac Radiation Exposure and the Risk of Arrhythmia in Breast Cancer Patients Treated With Radiotherapy: A Case–Control Study

**DOI:** 10.3389/fonc.2022.892882

**Published:** 2022-07-04

**Authors:** Mohamed Yassir Errahmani, Médéa Locquet, Daan Spoor, Gaelle Jimenez, Jérémy Camilleri, Marie-Odile Bernier, David Broggio, Virginie Monceau, Jean Ferrières, Juliette Thariat, Serge Boveda, Youlia Kirova, Pierre Loap, Johannes A. Langendijk, Anne Crijns, Sophie Jacob

**Affiliations:** ^1^ Laboratory of Epidemiology, Institute for Radiation Protection and Nuclear Safety (IRSN), Fontenay-Aux-Roses, France; ^2^ University Paris-Saclay, Gif-sur-Yvette, France; ^3^ Department of Radiation Oncology, University Medical Center Groningen (UMCG), University of Groningen, Groningen, Netherlands; ^4^ Department of Radiation Oncology (Oncorad), Clinique Pasteur, Toulouse, France; ^5^ Department of Dosimetry, Institute for Radiation Protection and Nuclear Safety (IRSN), Fontenay-Aux-Roses, France; ^6^ Laboratory of Radiotoxicology and Radiobiology, Institute for Radiation Protection and Nuclear Safety (IRSN), Fontenay-Aux-Roses, France; ^7^ Department of Cardiology and INSERM UMR 1295, Rangueil University Hospital, Toulouse, France; ^8^ Department of Radiotherapy, Centre de Lutte Contre le Cancer A. Baclesse, University of Caen Normandie, Caen, France; ^9^ Heart Rhythm Management Department, Clinique Pasteur, Toulouse, France; ^10^ Department of Radiation Oncology, Institut Curie, Paris, France

**Keywords:** breast cancer, radiation therapy, cardiotoxicity, cardiac arrhythmia, cardiac dosimetry

## Abstract

**Background:**

Previous studies suggested that radiation therapy (RT) for breast cancer (BC) can induce cardiac arrhythmias and conduction disorders. However, the association with mean heart dose and specific cardiac substructures doses was less studied.

**Materials and Methods:**

We conducted a nested case–control study based on French BC patients, enrolled in the European MEDIRAD-BRACE study (https://clinicaltrials.gov, Identifier: NCT03211442), who underwent three-dimensional conformal radiation therapy (3D-CRT) between 2009 and 2013 and were retrospectively followed until 2019. Cases were incident cases of cardiac arrhythmia. Controls without arrhythmia were selected with propensity-scored matching by age, duration of follow-up, chemotherapy, hypertension, and diabetes (ratio 1:4 or 5). Doses to the whole heart (WH), left and right atria (LA and RA), and left and right ventricles (LV and RV) were obtained after delineation with multi-atlas-based automatic segmentation.

**Results:**

The study included 116 patients (21 cases and 95 controls). Mean age at RT was 64 ± 10 years, mean follow-up was 7.0 ± 1.3 years, and mean interval from RT to arrhythmia was 4.3 ± 2.1 years. None of the results on association between arrhythmia and cardiac doses reached statistical significance. However, the proportion of right-sided BC was higher among patients with arrhythmia than among controls (57% vs. 51%, OR = 1.18, *p* = 0.73). Neither mean WH dose, nor LV, RV, and LA doses were associated with an increased risk of arrhythmia (OR = 1.00, *p* > 0.90). In contrast, the RA dose was slightly higher for cases compared to controls [interquartile range (0.61–1.46 Gy) vs. (0.49–1.31 Gy), *p* = 0.44], and a non-significant trend toward a potentially higher risk of arrhythmia with increasing RA dose was observed (OR = 1.19, *p* = 0.60). Subanalysis according to BC laterality showed that the association with RA dose was reinforced specifically for left-sided BC (OR = 1.76, *p* = 0.75), while for right-sided BC, the ratio of mean RA/WH doses may better predict arrhythmia (OR = 2.39, *p* = 0.35).

**Conclusion:**

Despite non-significant results, our exploratory investigation on BC patients treated with RT is the first study to suggest that right-sided BC patients and the right atrium irradiation may require special attention regarding the risk of cardiac arrhythmia and conduction disorders. Further studies are needed to expand on this topic.

## Introduction

Adjuvant radiation therapy (RT) after surgery is commonly used to treat localized breast cancer (BC) and generally results in significant improvement in tumor control and reduces the risk of cancer-related death several years after treatment (1, 2). However, BC survivors can develop a wide array of cardiotoxic complications related to cardiac radiation exposure, arising from a few months to many years after RT (3–5). Coronary artery disease is the most common manifestation of radiation‐induced cardiovascular disease and also the most described in the literature (5). A relative increase of 7.4% in lifetime risk of coronary events for each Gy (Gray) of radiation to the heart has been demonstrated in women with previous BC having received radiation (6), reaching 16.5% for the first 9 years after RT (6, 7). Such complications are more commonly seen in patients with left‐sided rather than right‐sided BC as a larger portion of the heart, in particular the left anterior descending artery, is included in the radiation field (6, 8).

Among cardiac complications of thoracic RT, arrhythmia and conduction disorders are much less frequent than coronary artery disease [approximately 4%–5% (9)] and investigations on these complications were limited. Several case reports suggested a link between RT for BC and atrioventricular nodal bradycardia, and all levels of heart block, including complete heart block and sick sinus syndrome (10–12). Some cohort studies have shown that BC patients treated with RT had a higher risk of morbidity and mortality of cardiac arrhythmia than BC patients not treated with RT (13, 14). More recently, patients with BC who have undergone RT have been shown to have a 2.2‐fold risk of conduction disorder requiring pacemaker implantation compared with the general population (15).

The question that remains is whether the risk of arrhythmia and conduction disorders, summarized under the general term “arrhythmia”, is related to cardiac exposure due to RT. There are distinct etiologies for different types of radiotherapy-associated cardiotoxicity, and the dose–response relationship previously observed between the mean heart dose and the coronary complications cannot be directly applied to arrhythmias. Very few studies have evaluated whether the risk of arrhythmia increases with mean heart dose and specific cardiac substructure doses. In a study performed on lung cancer patients treated with RT between 1996 and 2009, arrhythmic events showed borderline significant associations with the whole heart dose and right atrium doses, but not with left ventricle or left atrium doses (16). However, cardiac radiation exposure and dose distributions are very different according to the type of cancer treated and further studies remain needed, particularly for BC patients who have undergone RT.

In order to specifically investigate the potential relationship between cardiac exposure and the risk of arrhythmia in BC patients treated with RT, we conducted a study to assess the association with whole heart and cardiac substructure doses including left and right ventricles, and left and right atria.

## Materials and Methods

### Study Population

The nested case–control study was based on the French subgroup of left- and right-sided BC patients (*n* = 347) included in the multicenter MEDIRAD BRACE study further detailed elsewhere (ClinicalTrials.gov Identifier: NCT03211442).

The study population was composed of female patients, aged 40–75 years, who had undergone radiotherapy (3D-CRT) for a histologically proven diagnosis of BC (invasive and *in situ*) at Clinique Pasteur in Toulouse between January 2009 and December 2013.

After the surgical treatment of BC, all patients were treated with 3D-CRT with 6 and 25 MV photon beams by tangential fields, possibly including regional lymph node irradiation (internal mammary chain and supra-infraclavicular lymph nodes). The planning target volume dose was mostly 50 Gy delivered in 25 daily fractions of 2 Gy over 5 weeks or less frequently 32.5 Gy delivered in 5 daily fractions of 6.5 Gy. For most patients, 6 MV photons were used, except for a few cases of patients with big breast where 25 MV additional photons were used. An additional boost of 9–15 Gy could be applied to the tumor site using photon/electron beams with energies ranging from 6 MeV to 18 MeV. The treatment planning system (TPS) used to perform dose calculations was Eclipse™ with the Analytical Anisotropic Algorithm (AAA v13.6) (Varian Medical System, Palo Alto, CA, USA). Each patient’s RT was planned such that the dose distribution was optimized and normalized to the International Commission on Radiation Units and Measurements (ICRU) reference point of the breast and to achieve QUANTEC dose constraints to organs at risk including the heart (17), and deep-inspirational breath-hold (DIBH) technique was used for very few left-sided patients.

Patients’ characteristics at baseline including comorbidities and history of cardiac arrhythmia (conduction disorders or arrhythmia) were extracted from medical records of the Clinique, completed with medical records of patients’ general practitioners.

Patients with bilateral tumors, with distant metastasis at initial diagnosis, with previous RT before their initial BC treatment, with a history of cardiac arrhythmia, or without computed tomography for RT planning were excluded.

Follow-up data were retrospectively extracted from patients’ medical records from their general practitioners from the date of RT through July 2019. Based on these medical records, we defined incident arrhythmia cases as any conduction disorders or arrhythmia events recorded by patients’ general practitioners between the date of RT and July 2019.

To select controls corresponding to each arrhythmia case, propensity score matching with nearest-neighbor pairing was performed (18) based on factors known to potentially increase arrhythmia risk, including age at BC diagnosis, duration of follow-up (time from radiotherapy to the last observed follow-up time ≤ July 2019), the use of chemotherapy, and history of hypertension or diabetes. Laterality was not considered in propensity score matching in order to prevent overmatching between cases and controls regarding cardiac exposure. At least 4 or possibly 5 BC controls corresponding to each arrhythmia case were matched.

### Radiation Dosimetry

For all patients, cardiac structure delineation was performed by UMCG using multi-atlas-based automatic segmentation (MABAS). The whole heart (WH) and its substructures, including the left and right ventricle (LV and RV), and the left and right atria (LA and RA), were recontoured using the MABAS tool of the heart developed in-house based on the atlas by Feng et al. (19) (Mirada RTx [version 1.6]; Mirada Medical, Oxford, United Kingdom) (20). The exact planned radiation dose was reconstructed from the delineated volumes, and dose-volume histograms were obtained for each patient. In the current analysis, mean doses (Dmean, in Gy) to the whole heart and cardiac substructures were considered to evaluate the dose–response relationship with the risk of arrhythmia.

### Statistical Analysis

Conditional logistic regression, conditioned on the matching propensity score (including age, duration of follow-up chemotherapy, hypertension, and diabetes), was used to estimate the odds ratios of incident arrhythmia associated with clinical characteristics, BC laterality, and cardiac doses (for whole heart, left and right ventricles, and left and right atria). For cardiac doses, we analyzed Dmean as continuous variables, and evaluated the risk for higher doses (Dmean > 75th percentile) taking the group of patients with Dmean < 75th percentile as the reference group. Comparisons of doses according to BC laterality or case/control status were analyzed with paired Wilcoxon signed ranks tests. Spearman correlations were performed to evaluate the strength of the association between the whole heart dose and other cardiac substructure doses. Significance tests were two sided. A value of *p* < 0.05 was considered statistically significant. Statistical analysis was performed with the use of SAS Enterprise Guide version 4.3 and SAS version 9.4 (SAS Institute Inc, Cary, NC).

## Results

### Patient Characteristics

The study included 116 patients: 21 incident arrhythmia cases and 95 controls without arrhythmia (5 matching controls were found for 11 cases and 4 matching controls were found for the 10 other cases). Mean age at radiotherapy was 64 ± 10 years and mean follow-up corresponding to the time from radiotherapy to the last observed follow-up time (≤ July 2019) was 7.0 ± 1.3 years. The mean interval from radiotherapy to arrhythmia was 4.3 ± 2.1 years. Matched characteristics were similar in cases and controls (**Table 1**). We observed higher frequencies of mastectomy, hormonal therapy, smoking status, hypercholesterolemia, or dyslipidemia in cases compared to controls, with corresponding OR ranging from 1.23 for dyslipidemia to 2.03 for hormonal treatment, but none of these covariates reached statistical significance in OR evaluations. Despite a slightly higher frequency of right-sided BC among cases compared to controls (57% vs. 51%), right laterality could not be established as a statistically significant risk factor of arrhythmia [OR = 1.18 (95% CI 0.47–3.08), *p* = 0.73].

**Table 1:** Clinical characteristics at initiation of radiotherapy for BC and relative risk of arrhythmia.

Characteristics	Cases (*n* = 21)	Controls (*n* = 95)	Odds Ratio (95% CI)	*p*-value
**Matched characteristics**				
Age at diagnosis, in years	66.14 ± 10.95	64.06 ± 10.25	NA	NA
Follow-up, in years	6.99 ± 1.53	6.97 ± 1.76	NA	NA
Chemotherapy, *N* (%)				
No	15 (71.43)	64 (67.37)	NA	NA
Yes	6 (28.57)	31 (32.63)		
Hypertension, *N* (%)				
No	8 (38.10)	40 (42.11)	NA	NA
Yes	13 (61.90)	55 (57.89)		
Diabetes, *N* (%)				
No	20 (95.24)	92 (96.84)	NA	NA
Yes	1 (4.76)	3 (3.16)		
**Other characteristics**				
Type surgery, *N* (%)				
Lumpectomy	18 (85.71)	87 (92.55)	1.00	0.37
Mastectomy	3 (14.29)	7 (7.45)	1.97 (0.45–8.70)	
Hormonal therapy, *N* (%)				
No	4 (19.05)	30 (31.58)	1.00	0.25
Yes	17 (80.95)	65 (68.42)	2.03 (0.61–6.78)	
Smoke, *N* (%)				
No	18 (85.71)	83 (87.37)	1.00	0.75
Yes	3 (14.29)	12 (12.63)	1.27 (0.29–5.67)	
Hypercholesterolemia, *N* (%)				
No	9 (42.86)	50 (52.63)	1.00	0.55
Yes	12 (57.14)	45 (47.37)	1.35 (0.51–3.58)	
Dyslipidemia, *N* (%)				
No	11 (52.38)	56 (58.95)	1.00	0.67
Yes	10 (47.62)	39 (41.05)	1.23 (0.47–3.23)	
**Laterality of BC**				
Left-sided BC, *N* (%)	9 (42.86)	47 (49.47)	1.00	
Right-sided BC, *N* (%)	12 (57.14)	48 (50.53)	1.18 (0.46–3.04)	0.73

95% CI: 95% Confidence Interval. "NA" for "Not Applicable".

### Whole Heart and Cardiac Substructure Dosimetry

**Figure 1:**
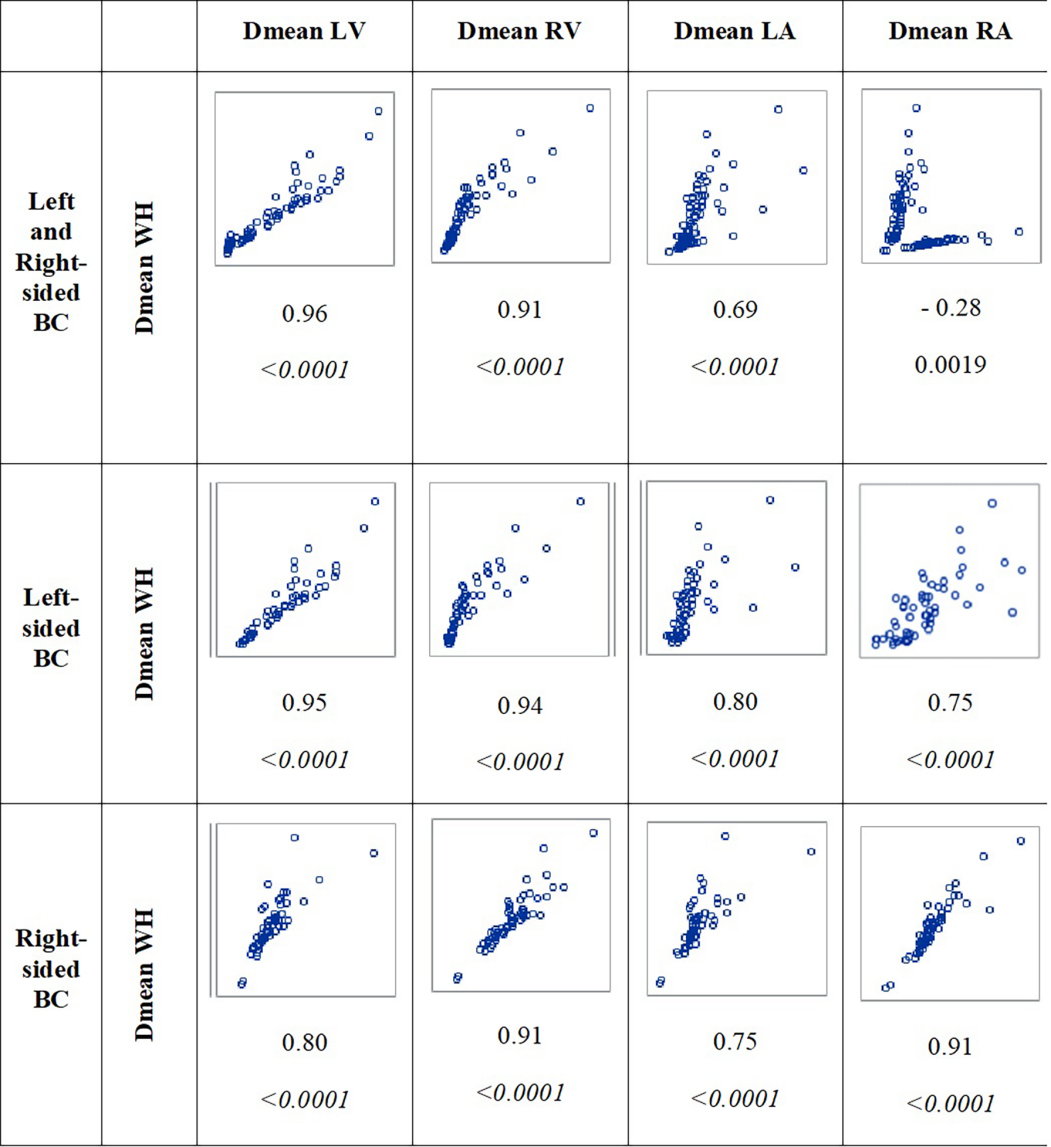
Correlations between whole heart dose (Dmean WH) and cardiac substructure doses (left ventricle, right ventricle, left atrium, and right atrium) for all patients and according to BC laterality. *r* and the corresponding *p*-value are indicated below the graphs.

Median mean dose to the whole heart was 0.97 Gy, with higher doses for left-sided BC compared to right-sided BC (3.38 Gy vs. 0.59 Gy, *p* < 0.0001). Similar findings were observed for the left ventricle, right ventricle, and left atrium, with higher doses for left-sided BC (**Table 2**). In contrast, for the right atrium, doses were significantly higher for right-sided BC compared to left-sided BC (1.33 Gy vs. 0.50 Gy, *p* < 0.0001). The correlations of the whole heart dose with cardiac substructure doses were high for the left and right ventricles and the left atrium, independently from BC laterality (**Figure 1**). For the right atrium, the correlation with the whole heart dose showed a spurious negative association (*r* = −0.28) resulting from differences according to laterality of BC: the group of patients with a lower whole heart dose (corresponding to right-sided BC) could receive high right atrium doses whereas the group of patients with a high whole heart dose (corresponding to left-sided BC) received lower right atrium doses. Subanalysis of correlation between the whole heart and right atrium doses according to BC laterality showed a positive correlation for left-sided BC (*r* = 0.75) and right-sided BC (*r* = 0.91). In order to consider the right atrium dose according to the whole heart dose, we analyzed the ratio Dmean RA/Dmean WH, which showed that the right atrium dose was 0.17 times lower than the whole heart dose for left-sided BC and 2.42 times higher than the whole heart dose for right-sided BC (**Table 3**).

**Table 2:** Whole heart and cardiac substructure doses for all patients and according to BC laterality.

	All patients (*N* = 116) Median (interquartile range)Min–Max	Left-sided BC (*n* = 56) Median (interquartile range)Min–Max	Right-sided BC (*n* = 60) Median (interquartile range)Min–Max	*p*-value Left vs. Right
**Dmean Whole Heart, Gy**	0.97 (0.58–3.30) 0.0021–11.47	3.38 (1.56–4.80) 0.79–11.47	0.59 (0.46–0.71) 0.0021–1.48	<0.0001
**Dmean Left Ventricle, Gy**	0.41 (0.16–4.33) 0.0009–13.22	4.47 (1.99–6.61) 1.01–13.22	0.15 (0.11–0.21) 0.0009–0.77	<0.0001
**Dmean Right Ventricle, Gy**	0.95 (0.61–2.24) 0.0012–18.96	2.45 (1.25–4.49) 0.69–18.96	0.62 (0.44–0.78) 0.0012–1.59	<0.0001
**Dmean Left Atrium, Gy**	0.51 (0.39–0.76) 0.0028–3.69	0.70 (0.55–0.92) 0.22–3.69	0.41 (0.36–0.50) 0.0028–1.76	<0.0001
**Dmean Right Atrium, Gy**	1.00 (0.49–1.34) 0.0024–4.21	0.50 (0.34–0.60) 0.13–1.27	1.33 (1.14–1.64) 0.0024–4.21	<0.0001

Dmean, mean dose.

**Table 3:** Ratio Dmean RA/Dmean WH for all patients and according to BC laterality.

	RatioDmean RA/Dmean WH		
	Mean ± SD	Median	Min–Max
**All patients**	1.34 ± 1.17	1.75	0.07–4.06
**Left-sided BC**	0.17 ± 0.08	0.15	0.07–0.36
**Right-sided BC**	2.42 ± 0.42	2.36	1.14–4.05

### Impact of Cardiac Doses on the Risk of Arrhythmia

**Figure 2:**
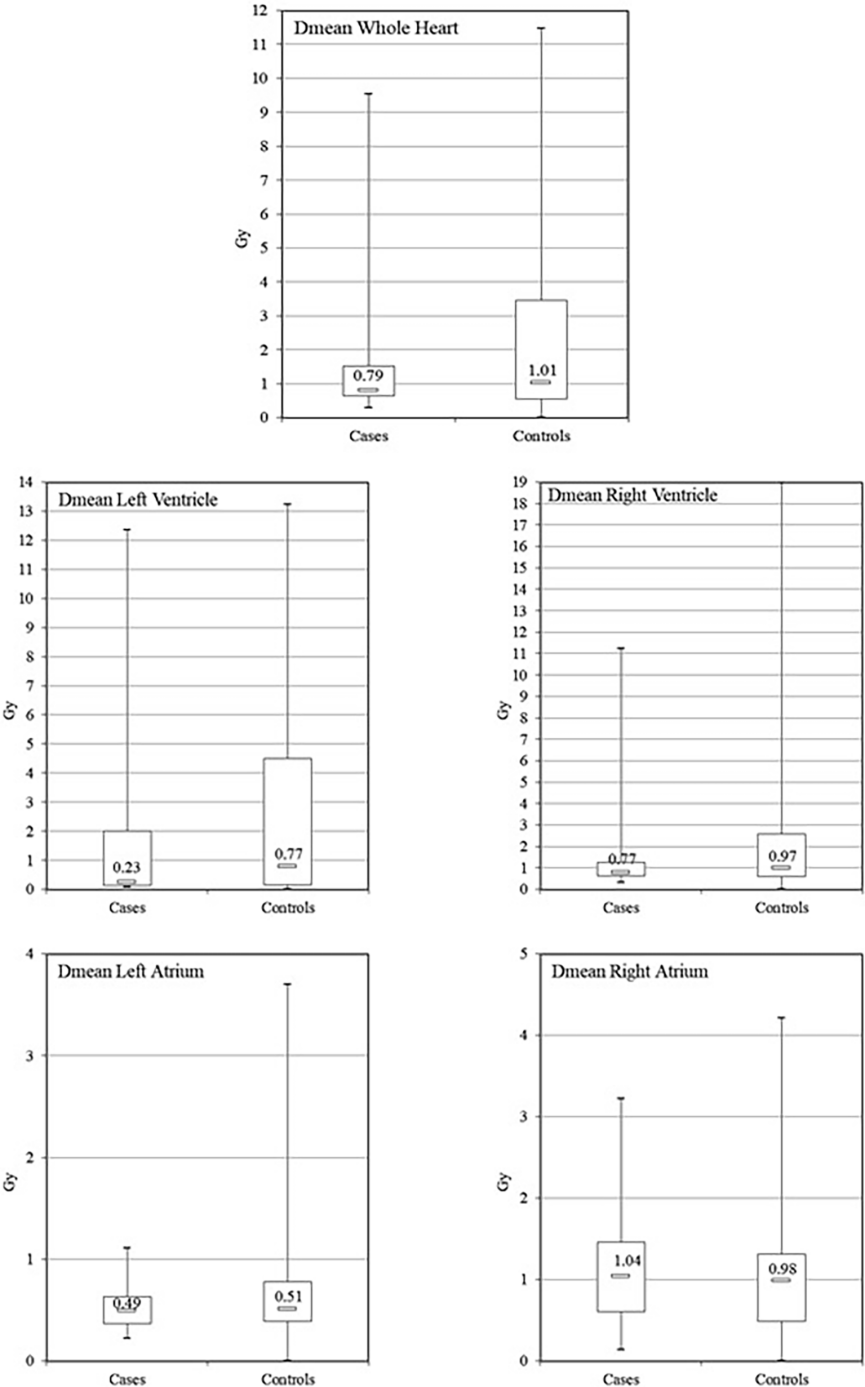
Comparison of mean dose distribution for whole heart and cardiac substructures according to case or control status. [The central value of the box indicates the median, the borders of the box indicate the quartiles (25th and 75th), and the extremities indicate the minimum and maximum values.

Our study was limited in size, and none of the results presented here reached statistical significance. However, there are several findings. For the whole heart, left and right ventricles, and left atrium, lower doses were observed for cases compared to controls, without reaching statistical significance (**Figure 2**). However, for the right atrium, mean doses were slightly higher for cases than controls (cases: median = 1.04 Gy, interquartile range = 0.61–1.46; controls: median = 0.98 Gy, interquartile range 0.49–1.31; *p* = 0.44). As previously indicated, cases with arrhythmia were more likely to be right-sided BC than controls, without reaching statistical significance [OR = 1.18 (0.46–3.04)]. The frequency of patients receiving high doses (corresponding to doses > 75th percentile of dose distribution) to the whole heart, left and right ventricles, and left atrium (>3.30 Gy, 4.33 Gy, 2.24 Gy, and 0.76 Gy, respectively) was lower among cases than among controls (19% vs. 26%), yielding an OR < 1 but not statistically significant. Moreover, the risk of arrhythmia did not increase with increasing mean dose to the whole heart [OR = 1.00 (0.81–1.25), *p* = 0.98], and similar findings were observed for left and right ventricles and left atrium doses (OR = 1.00, 1.00, and 0.54, respectively) (**Table 4**). In contrast, the frequency of patients receiving high doses to the right atrium (>1.34 Gy) was higher among cases than among controls (33% vs. 22%), yielding an OR = 1.50 (0.58–3.88), *p* = 0.39, not statistically significant. A non-significant trend toward a potentially higher risk of arrhythmia with increasing mean dose to the right atrium was observed [OR = 1.19 (0.63–2.23), *p* = 0.60]. Subanalysis according to BC laterality (**Table 5**) showed that for left-sided BC, despite being non-significant, a potential association with the right atrium dose was observed (OR = 1.76, *p* = 0.75), as well as with the whole heart dose (OR = 1.28, *p* = 0.26). For right-sided BC, no direct association with RA dose alone was observed, but the ratio of mean doses RA/WH may have potential to predict arrhythmia (OR = 2.39, *p* = 0.35).

**Table 4:** Association between cardiac doses and risk of arrhythmia: continuous trend and values > 75th percentile of dose distribution.

Laterality or cardiac doses	% cases vs.% controls	Odds Ratio* (95% CI)	*p*-value
**Right-sided BC** ^a^	57% vs. 51%	1.18 (0.46–3.04)	0.73
**Whole Heart**			
Dmean, in Gy	–	1.00 (0.81–1.25)	0.98
Dmean > 3.30 Gy ^b^	19% vs. 26%	0.76 (0.23–2.49)	0.66
**Left Ventricle**			
Dmean, in Gy	–	1.00 (0.86–1.17)	0.98
Dmean > 4.33 Gy ^c^	19% vs. 26%	0.77 (0.24–2.84)	0.65
**Right Ventricle**			
Dmean, in Gy	–	1.01 (0.86–1.19)	0.91
Dmean > 2.24 Gy ^d^	19% vs. 26%	0.76 (0.23–2.55)	0.66
**Left Atrium**			
Dmean, in Gy	–	0.54 (0.14–2.08)	0.37
Dmean > 0.76 Gy ^e^	19% vs. 26%	0.70 (0.22–2.24)	0.54
**Right Atrium**			
Dmean, in Gy	–	1.19 (0.63–2.23)	0.60
Dmean > 1.34 Gy ^f^	33% vs. 22%	1.50 (0.58–3.88)	0.39

*Odds ratios are unadjusted as none of the baseline characteristics in **Table 1** reached p-value < 0.20; CI: confidence interval. ^a^Left-sided BC as reference category; ^b^Dmean ≤ 3.30 Gy as reference category; ^c^Dmean ≤ 4.33 Gy as reference category; ^d^Dmean ≤ 2.24 Gy as reference category; ^e^Dmean ≤ 0.76 Gy as reference category; ^f^Dmean ≤ 1.34 Gy as reference category. BC, breast cancer; Dmean, mean dose; WH, whole heart; RA, right atrium.

**Table 5:** Subgroup (left-sided BC and right-sided BC) analysis of the association between risk of arrhythmia and whole heart and right atrium doses.

	Odds Ratio*(95% CI)	*p*-value
**Left-sided BC**		
Dmean WH, in Gy	1.28 (0.83–1.98)	0.26
Dmean RA, in Gy	1.76 (0.05–59.54)	0.75
Ratio Dmean RA/Dmean WH	0.90 (0.0–na)	0.99
**Right-sided BC**		
Dmean WH, in Gy	0.33 (0.02–5.20)	0.43
Dmean RA, in Gy	0.90 (0.30–2.25)	0.70
Ratio Dmean RA/Dmean WH	2.39 (0.38–14.90)	0.35

*Odds ratios are unadjusted as none of the baseline characteristics in **Table 1** reached p-value < 0.20; CI: confidence interval; BC, breast cancer; Dmean, mean dose; WH, whole heart; RA, right atrium.

## Discussion

This exploratory study on the association between cardiac exposure and the risk of arrhythmia in BC patients treated with RT has several important findings. First, regarding the whole heart and the four cardiac chamber doses, the right atrium dose is the only structure presenting higher doses for right-sided BC compared to left-sided BC. Patients with arrhythmia were more likely to have right-sided BC than patients without arrhythmia. This could be explained by higher doses to the right atrium for right-sided BC. Second, an increased risk of arrhythmia was suggested with increasing dose to the right atrium, either directly, for patients with left-sided BC, or proportionally to the whole heart dose, for patients with right-sided BC. These results illustrate the potential relevance of right atrium exposure regarding the risk of arrhythmia.

Cardiac arrhythmias and bradycardia including conduction disorders are a broad category of potential complications of radiotherapy for BC as observed in previous studies. In a cohort of 746 BC patients, the cumulated incidence at 10 years of arrhythmia/conduction disorder reached 4.8% in patients treated with RT versus 0% in patients not treated with RT (14). In this study, the definition of the event was broadly defined by arrhythmia/conduction disorder without additional information. In a French study based on a national healthcare database including a group of 2,973 BC patients treated with RT between 2008 and 2016 with a mean follow-up of 6 years, 28 cases of *de novo* pacemaker implantation were observed, which was 2.18 times higher than expected in the general population, whereas in the group not treated with RT, the number of pacemaker implantation was similar to the one expected in the general population (15). However, the relationship between the occurrence of these events and the level of cardiac radiation exposure has been sparsely studied.

There are distinct etiologies for different types of radiotherapy-associated cardiotoxicity, and the whole heart dose may not be the best predictor of all types of radiation-related heart disease (21). The occurrence of cardiac arrhythmias and conduction disorders after RT is usually associated with fibrosis, which might lead to alteration of conduction pathways with associated fibrosis of nodal structures (sinus and atrioventricular nodes) leading to rhythm changes (11). The tissue fibrosis induced by RT could be responsible for non-specific secondary cardiac lesions at the atrial, ventricular, and coronary levels, which are the basis for arrhythmias and bradycardia. However, the relatively low radiation doses and short follow-up in our study may not have caused significant cardiac radiation fibrosis. The sino-atrial node is located in the wall (epicardium) of the right atrium. The compact atrioventricular node is also located in the right atrium, close to the interatrial septum and the coronary sinus ostium. As a consequence, the right atrium dose may be a relevant proxy of these nodes’ dosimetry and relevant for atrial arrhythmia and conduction disorders. In our study, we observed that right-sided irradiation was associated with a greater exposure to the right atrium, in contrast with other cardiac substructures, as previously observed (22). The analysis of the correlation between the whole heart dose and cardiac substructure doses showed strong correlation between the whole heart dose and left and right ventricle doses (left ventricle: *r* = 0.95 for left-sided BC and slightly lower *r* = 0.80 for right-sided BC; right ventricle: *r* = 0.94 for left-sided BC and 0.91 for right-sided BC). Similar findings were observed previously for whole heart and left ventricle doses with *r* = 0.78 for left-sided BC and lower *r* = 0.55 for right-sided BC (23). However, we found a moderate correlation between whole heart and right atrium doses for left-sided BC patients (*r* = 0.75), illustrating that the mean heart dose may not be a good surrogate parameter of the right atrium dose, prompting a specific investigation of this cardiac substructure exposure regarding the risk of arrhythmia.

Some previous studies have investigated the relationship between doses to particular cardiac substructures and subsequent damage to those structures in BC patients, relating coronary artery doses to subsequent coronary artery stenosis (24) or left ventricle dose to subclinical left ventricular abnormalities (25, 26). In our study, the results suggested that the right atrium dose may be a better predictor of arrhythmia than the whole heart dose. For right-sided BC, the mean right atrium dose/mean heart dose ratio may be an interesting predictor of arrhythmia (OR = 2.39, *p* = 0.35). Such association with right atrium exposure would indeed be more relevant for atrial arrhythmias or conduction disorders more than ventricular arrhythmias, but this could not be checked in our data with no specific detail on arrhythmia/conduction disorders. Despite differences in the type of cancer treated and consequently level of cardiac exposure during cancer treatment, the association between right atrium dose and the risk of arrhythmia was previously presented in a cohort of 112 lung cancer patients treated with radiotherapy, followed on average for 9 years, among whom 12 arrhythmic events had been identified. Similarly to our study, no details on the type of arrhythmic event were provided, and this study, despite the fact that lung cancer RT led to higher radiation doses to the heart than in the current BC study, showed a relatively weak association with the whole heart dose (HR = 1.02, *p* = 0.054) and right atrium doses (HR = 1.02, *p* = 0.054), but not with the left ventricle or left atrium doses (16).

Studies that have analyzed the impact of cardiac dosimetry on potentially critical substructures for arrhythmias such as the sino-atrial node or the atrioventricular node are rare (27, 28). The prominent pacemaker role of the right atrium nodes should be kept in mind, and arrhythmia might be a long-term cardiac adverse event to consider specifically for right-sided BC patients. These nodal structures can be located, with some uncertainty, on the RT computed tomography and could therefore provide information on the association between cardiac exposure and the risk of atrial arrhythmias and conduction disorders (29).

We acknowledge that this study has some limitations. First, the size of the sample was small and the duration of follow-up was not very long, resulting in low statistical power, which could explain the absence of statistical significance in results. However, this study was exploratory, and its results encourage researchers to further analyze the risk of arrhythmia in larger studies. The definition of the arrhythmia event was based on medical records from BC patients’ general practitioners. Despite the intensive work to collect data through general practitioners, we cannot be sure that the identification of incident cases of arrhythmia was complete. Consequently, some arrhythmia events among selected controls may be present. Such possible classification bias would nevertheless tend to dilute our results and not yield to spurious enhanced risk related to dose. Furthermore, no detail on the type of arrhythmia (atrial, ventricular, and conduction disorder) was provided, which may be of interest for better knowledge on the occurrence of these events. Whole heart and cardiac substructure dosimetry was based on MABAS. Such methodology was previously validated, showing reliability and efficiency for contouring of cardiac substructures and obtaining cardiac dose parameters with accuracies at least similar to interobserver delineation variation (20). However, such methodology may, in some patients, be less precise than manual segmentation, involving possible inaccuracies in the doses and resulting in “noise” in the evaluation of dose–response relationship. None of the potential risk factors of arrhythmia, including hormonal therapy for example (OR = 2.03, *p* = 0.25), reached statistical significance, which may mainly reflect an under power of our study as stated above. Hormonal therapy combined with radiotherapy may increase the risk of arrhythmia (30), but this could not be evaluated in our study. Cardiac radiation doses in BC therapy are low compared to certain other thoracic cancer therapies that were previously considered in previous studies such as lung cancer, resulting in lower dose ranges and issues that highlight the potential RT-associated risk. Another limitation is the use of old techniques of irradiation in our study. DIBH can substantially decrease cardiac exposure in patients (22), and intensity-modulated radiotherapy with helical tomotherapy has shown a very low rate of cardiac complications (31).

Further studies remain needed in order to enhance knowledge on the risk of arrhythmia and conduction disorder after RT for BC and refine the potential relationship between the risk of arrhythmia and exposure to cardiac substructures and, *in fine*, identify “high risk” patient profiles. These studies will require a larger number of patients, inclusion of patients treated with modern techniques of RT (DIBH, IMRT, proton therapy, and other adapted techniques), detailed cardiac substructures, and nodal dosimetry. Collecting information on the kind of arrhythmia (atrial or ventricular arrhythmia, conduction disorder) is also an important perspective as there may be differences in radiation sensitivity of various cardiac tissues (ventricular and atrial myocardium, conductive tissue, nodes, etc.).

## Conclusion

Our exploratory study on the risk of cardiac arrhythmia in BC patients treated with RT suggested that right-sided BC patients may require particular attention and the dose to the right atrium may be a more relevant dosimetry parameter than the whole heart or other cardiac substructure doses regarding the risk of arrhythmia. Such findings may be related to the location of the sino-atrial node (pacemaker cells) and conductive tissue in the right atrium and prompt researchers to further investigate more specifically these structures’ dosimetry.

## Data Availability Statement

The raw data supporting the conclusions of this article will be made available by the authors, on reasonable request.

## Ethics Statement

The study was subject to a declaration of compliance with a reference methodology concerning research in the field of health (MR 03) from the French Commission Informatique et Liberté - CNIL, (ref 2103119, September 27, 2017). The patients/participants provided their written informed consent to participate in this study.

## Author Contributions

All authors contributed to the study conception and design. Material preparation was carried out by ME, ML, DS, JAL, AC, and SJ. Data collection and analysis were performed by MYE, ML, DS, GJ, JC, MOB, AC, and SJ. All authors participated in the writing of the manuscript. All authors read and approved the final manuscript.

## Funding

This work was funded by H2020 Euratom research and training program 2014–2018 under grant agreement No. 755523 in the frame of the MEDIRAD project.

## Conflict of Interest

The authors declare that the research was conducted in the absence of any commercial or financial relationships that could be construed as a potential conflict of interest.

The reviewer MB is currently organizing a Research Topic with the author VM.

## Publisher’s Note

All claims expressed in this article are solely those of the authors and do not necessarily represent those of their affiliated organizations, or those of the publisher, the editors and the reviewers. Any product that may be evaluated in this article, or claim that may be made by its manufacturer, is not guaranteed or endorsed by the publisher.
